# GWAS findings improved genomic prediction accuracy of lipid profile traits: Tehran Cardiometabolic Genetic Study

**DOI:** 10.1038/s41598-021-85203-8

**Published:** 2021-03-11

**Authors:** Mahdi Akbarzadeh, Saeid Rasekhi Dehkordi, Mahmoud Amiri Roudbar, Mehdi Sargolzaei, Kamran Guity, Bahareh Sedaghati-khayat, Parisa Riahi, Fereidoun Azizi, Maryam S. Daneshpour

**Affiliations:** 1 Cellular and Molecular Research Center, Research Institute for Endocrine Sciences, Shahid Beheshti University of Medical Sciences, POBox: 19195-4763, Tehran, Iran; 2 Department of Animal Science, Safiabad-Dezful Agricultural and Natural Resources Research and Education Center, Agricultural Research, Education & Extension Organization (AREEO), Dezful, Iran; 3 Department of Pathobiology, Ontario Veterinary College, University of Guelph, Guelph, Canada; 4 Select Sires Inc., Plain City, USA; 5 Endocrine Research Center, Research Institute for Endocrine Sciences, Shahid Beheshti University of Medical Sciences, Tehran, Iran

**Keywords:** Computational biology and bioinformatics, Developmental biology, Genetics

## Abstract

In recent decades, ongoing GWAS findings discovered novel therapeutic modifications such as whole-genome risk prediction in particular. Here, we proposed a method based on integrating the traditional genomic best linear unbiased prediction (gBLUP) approach with GWAS information to boost genetic prediction accuracy and gene-based heritability estimation. This study was conducted in the framework of the Tehran Cardio-metabolic Genetic study (TCGS) containing 14,827 individuals and 649,932 SNP markers. Five SNP subsets were selected based on GWAS results: top 1%, 5%, 10%, 50% significant SNPs, and reported associated SNPs in previous studies. Furthermore, we randomly selected subsets as large as every five subsets. Prediction accuracy has been investigated on lipid profile traits with a tenfold and 10-repeat cross-validation algorithm by the gBLUP method. Our results revealed that genetic prediction based on selected subsets of SNPs obtained from the dataset outperformed the subsets from previously reported SNPs. Selected SNPs’ subsets acquired a more precise prediction than whole SNPs and much higher than randomly selected SNPs. Also, common SNPs with the most captured prediction accuracy in the selected sets caught the highest gene-based heritability. However, it is better to be mindful of the fact that a small number of SNPs obtained from GWAS results could capture a highly notable proportion of variance and prediction accuracy.

## Introduction

It raised an enormous possibility of predicting complex phenotypes from genotypes as the initial results of the human genome project's sequence were publicly available^1^. Our understanding of the human genome can be applied to improve personal medicine to prevent diseases, diagnosis, and treatment. Hence, it has enriched health care from birth through life^2,3^. We can also classify individuals into various susceptibility levels of complex disease by utilizing genetic testing and have earmark resources for public health research that results in targeted treatment through pharmacogenomics. Recent promising discoveries from Genome-Wide Association Studies (GWASs) have provided insight into clinical applications^4^. GWASs have mainly discovered and reported several significant Single Nucleotide Polymorphisms (SNPs) associated with various types of human complex traits and diseases (e.g., GWAS Catalog^5^). However, even in highly heritable phenotypes, the combination of significantly associated SNPs' effects explains a small proportion of phenotypic variation^4,6^ and may not be sufficient to predict complex traits. To solve this problem, the idea of applying whole-genome Regression models (WGR) was presented to improve the accuracy of Genomic Prediction^7^ to capture the possible portion of phenotypic variation explained by the genome^8^. The Genomic Best Linear Unbiased Prediction (gBLUP) approach introduced by VanRaden and Habier^9,10^, is designed to estimate genetic values. This method employs Genomic Relationship Matrix (GRM) that improves genomic similarities between individuals^8,10,11^. Although the accuracy of the genetic prediction increases by using whole-genome information, there are still variants in the genome with small contributions to prediction. Thus, removing them would have no significant implication. Indeed, they are neither strong enough to have significant associations individually nor have their aggregation effect significantly impacted genetic prediction accuracy. It has been shown that although variable selection or shrinkage estimation procedure can handle the problem of the small contribution of SNPs, choosing an appropriate method for the preselection of SNPs can improve prediction ability^12^.

In this study, we aimed to incorporate the strength of both WGR and GWAS to find the optimized number of SNPs that have the most contribution to the explanation of genomic phenotypic variation and make GRM perform computationally efficient in gBLUP, using GCTA software^13^. Finally, the strategies are tested on lipid profile traits, including high-density lipoprotein cholesterol (HDL-C), low-density lipoprotein cholesterol (LDL-C), triglycerides (TG), and cholesterol (CHOL) extracted from Tehran Lipid and Glucose Study (TLGS) and Tehran Cardiometabolic Genetic Study (TCGS) projects^14^. Furthermore, we evaluated the strength of selected subsets of SNPs to explain the genotypic variance of lipid profile traits. We estimated gene-based heritability, which we declare this is the first report of gene-based heritability of lipid profile traits in the Iranian population.

## Method and materials

### Study population

Tehran Lipid and Glucose Study (TLGS), the first ongoing periodic cohort study of the Iranian population project, includes pedigrees of 1 to 38 members with an average number of 4.23 ± 4.11 individuals, age ranged from 3 to 80 years. For over 25 years, TLGS has provided a wide variety of epidemiological data. Non-communicable disorders’ (NCDs) risk factors of 15,000 participants have been recorded every three years. We have extracted the fourth phase's information of participants due to the availability of the most recorded information on lipid profile traits. The Tehran Cardiometabolic Genetic Study (TCGS) project was derived from TLGS, which provided most of the primitive study participants, 14,827 individuals, with more than 649,932 genetic variants.

All participants were requested to sign an informed written consent. The ethical committee of the Research Institute for Endocrine Sciences, Shahid Beheshti University of Medical Sciences, approved the design of the TLGS.

### Phenotype measurement

The TCGS participants with recorded lipid profile traits, including 10,301 people with HDL-C, 10,586 people with LDL-C, 10,303 people with TC, and 10,303 people with TG data, have been extracted (where the LDL-C was measured as LDL-C = TC − HDL −  (TG/5)). It should be noted that TG was in its log-transformed form to adjust for its highly skewed distribution. Based on the previous studies, we extract body mass index (BMI), age, and sex as covariates.

### Genotyping, quality control, and missing imputation

Blood samples of TCGS participants were genotyped using humanOmniExpress-24-v1 bead chips, which have provided us with 649,932 single nucleotide polymorphism loci with an average mean distance of 4 kilobases for each individual at deCODE genetic company as described comprehensively in^14^. At the beginning of our analysis of the genomic dataset, we needed to perform quality control (QC) based on both individuals and markers using plink software^15^. The steps are summarized in Supplementary Fig. 1. Before taking regular QC steps, we have implemented pedigree and parentage checks. We used S.A.G.E (Statistical Analysis for Genetic Epidemiology) software version 6.4^16^, the ped-info command, for the pedigree check to find any problem with recorded parental information. Next, we applied snp1101 software for checking contradictory information based on recorded parental and genotype platforms’ information^17,18^. 132 individuals had inconsistencies in their parental information, and we decided to consider them as a founder instead of being in a family structure.

Then we started individuals' and markers' QC using Plink software. First, we filtered SNPs and individuals with more than 0.2 missing rates (for both individuals and SNPs). This non-strict threshold was adopted to remove any low-quality SNPs and individuals in the dataset (770 SNPs and 11 individuals were removed at this step). Second, we made our filtering tighter. We applied the 0.02 threshold to exclude SNPs and individuals with less than 0.02 call rates (17,636 SNPs and no one was removed). Third, individuals with discrepancies in their recorded sex and gender determination were eliminated based on the X chromosome (no sex discrepancy was observed). Fourth, to maintain the study's power, it is recommended to ignore SNPs with low minor allele frequency (MAF), e.g., rare variants. The SNPs with MAFs lower than 0.05 were removed (72,500 SNPs were excluded). Next, markers that deviated from the Hardy–Weinberg equilibrium (HWE) assumption were excluded by the p-value of 1e−6 (1125 SNP markers were removed). Next, individuals who deviated from ± 3SD samples' heterozygosity rate mean were removed (317 individuals were removed). Finally, we checked for population stratification using principal component analysis (PCA) via R software's SNPRelate package^19^. After pruning for the (first/second) principal components via the multi-dimensional scaling method, the PCA plots are shown in Supplementary Fig. 2. The PCA plot reveals that subjects in a group are genetically similar to each other than another group. We captured the population stratification by entering 20 PCAs into the GWAS models. After all QC steps procedure, we used beagle 5.1 (version: 18May20.d20) software to impute missing genotypes^20^. Ultimately, the analysis was implemented on 13,785 individuals with 546,339 genetic markers.

### Statistical analysis

#### Model selection

We have applied multiple linear regression model, including age, sex, and BMI, as fixed factors for lipid profile traits. The stepwise approach, which is a combination of the forward and backward selection, considered all three above covariates to be included in the predictor model for HDL-C, LDL-C, TC, and log transformation of TG (to control high skewness). Therefore, the phenotype prediction study has been done with SNP markers as random effects and age, sex, BMI, and the first 20 principal components as fixed effects.

#### GBLUP

A mixed model was used as:

1 $${\varvec{y}}={\varvec{X}}{\varvec{\beta}}+{\varvec{Z}}{\varvec{u}}+{\varvec{\varepsilon}},$$

where ${\varvec{y}}$ is defined as the vector of observed phenotypes, ${y}_{i}$, with $i=1,\dots ,n$ ($n$ = number of subjects), ${\varvec{\beta}}$ indicates the vector of fixed effects (age, sex, and BMI), X is a design matrix relating the fixed effects to each individual, $u\sim N(0,{\varvec{I}}{\sigma }_{u}^{2})$ indicates a vector of SNP effects with a variance of ${\sigma }_{u}^{2}$, **I** is a square $n\times n$ identity matrix. ${\varvec{\varepsilon}}\sim N(0,{\sigma }_{\varepsilon }^{2})$ is the residual vector where ${\sigma }_{\varepsilon }^{2}$ indicates the variance of residuals. **Z** is a matrix of genotypes that indicates the number of reference allele copies (coded as 0,1and 2). If we transform the matrix **Z** to its standardized form, noted by **W**, we would have the following equation:

2 $${\varvec{y}}={\varvec{X}}{\varvec{\beta}}+{\varvec{W}}{\varvec{u}}+{\varvec{\varepsilon}},$$

with the variance of

$${\varvec{v}}{\varvec{a}}{\varvec{r}}({\varvec{y}})={\varvec{W}}{{\varvec{W}}}^{\boldsymbol{^{\prime}}}{{\varvec{\sigma}}}_{{\varvec{u}}}^{2}+{\varvec{I}}{{\varvec{\sigma}}}_{{\varvec{\varepsilon}}}^{2},$$

in which **W** is a matrix that its $ij{\text{th}}$($i{\text{th}}\;{\text{individual}} \;{\text{and}}\; j{\text{th}}$ SNP) element is ${w}_{ij}=({z}_{ij}-{2p}_{j})/\sqrt{{2p}_{j}(1-{p}_{j})}$, that ${p}_{j}$ shows the frequency of $j{\text{th}}$ SNP (j = 1, …, k). Regarding our objectives, which is the aggregation of SNPs' effects on the phenotype, if we define n × 1 vector of **g** total genetic effects of the individuals, we have the Eq. (2) mathematically equal to:

3 $${\varvec{y}}={\varvec{X}}{\varvec{\beta}}+{\varvec{g}}+{\varvec{\varepsilon}}$$

With the variance of.

$${\varvec{v}}{\varvec{a}}{\varvec{r}}({\varvec{y}})={\varvec{A}}{{\varvec{\sigma}}}_{{\varvec{g}}}^{2}+{\varvec{I}}{{\varvec{\sigma}}}_{\upvarepsilon }^{2}$$

Note that ${\varvec{A}}={\varvec{W}}{{\varvec{W}}}^{\boldsymbol{^{\prime}}}/{\varvec{K}}$ can be defined as the Genomic Relationship Matrix (GRM) between individuals. Based on the estimated GRM from entire SNPs, we can estimate the phenotypic variance explained by all the SNPs (${\sigma }_{g}^{2}$) as well as residual variance ${(\sigma }_{\varepsilon }^{2})$ by the restricted maximum likelihood (REML) method using GCTA software, which is applying the average information (AI) method to initiate its iterations.

Therefore, we can have the best linear unbiased prediction (BLUP) of the whole SNPs' effects for all individuals [$\widehat{{\varvec{g}}}$ in Eq. (3)]. Straightforwardly, we can have the estimation of each SNPs' effect based on Eqs. (2) and (3). In fact, having $\widehat{{\varvec{g}}}$, the BLUP of **u** ($\widehat{{\varvec{u}}})$ can be found with the following equation:

$$\widehat{{\varvec{u}}}={{\varvec{W}}}^{\boldsymbol{^{\prime}}}{{\varvec{A}}}^{-1}\widehat{{\varvec{g}}}/{\varvec{N}}$$

We know that ${\widehat{u}}_{j}$ is the coefficient of ${w}_{ij}$. So to have an estimation of SNP effect corresponded to ${z}_{ij}$ it is enough to transform it by ${\widehat{u}}_{j}^{*}={\widehat{u}}_{j}/\sqrt{{2p}_{j}(1-{p}_{j})}$. The BLUP effects that are achieved by GCTA can be used to gain the genetic value of the individuals for a given phenotype in a matched validation or test set, which means ${\widehat{g}}_{test}={w}_{test}\widehat{u}$. This feature provides us with the prediction of genetic value or an individual's risk to disease (polygenic risk score) in complex traits by using the PLINK version 1.9 scoring approach in a test dataset^15^.

### GRM calculation

Among various approaches that calculate GRM, in this study, we applied the method presented by Yang^8^. Genomic similarities between $i{\text{th}}$ and $i^{\prime}{\text{th}}$ individuals with entire SNPs can be defined as below. In the following formula ${A}_{i{i}^{^{\prime}}}$ indicates the similarity between $i{\text{th}}$ and $i^{\prime}{\text{th}}$ individuals in the $j{\text{th}}$ SNP, so with summation on $j$ we can capture the entire genomic resemblance between every two cases. Thus, when $i\ne i{^{\prime}}$:

$${A}_{i{i}^{^{\prime}}}=\frac{1}{k}\sum_{j=1}^{k}{A}_{ji{i}^{^{\prime}}}=\frac{1}{k}\sum_{j=1}^{k}\frac{({z}_{ij}-{2p}_{j})({z}_{i{^{\prime}}j}-{2p}_{j})}{{2p}_{j}(1-{p}_{j})}$$

Similarly, when $$i=i{^{\prime}}$$:

$${A}_{jk}=\frac{1}{k}\sum_{j=1}^{k}{A}_{ji{i}^{^{\prime}}}=1+\frac{1}{k}\sum_{j=1}^{k}\frac{{z}_{ij}^{2}-\left(1-{2p}_{j}\right){z}_{ij}+2{p}_{j}^{2}}{{2p}_{j}(1-{p}_{j})},$$

where ${z}_{ij}$ indicates the observed genotype of $j{\text{th}}$ SNP for $i{\text{th}}$ cases (coded as 0, 1, and 2 according to the number of copies of reference allele), and ${p}_{j}$ is the frequency of $j{\text{th}}$ SNP.

### Proposed SNP selection strategy

SNPs have been subsetted to calculate the GRM and have been applied for the subsequent prediction procedure based on GWAS results (considering 20 PCs) based on two viewpoints: First, the extraction of previously reported SNPs in association studies for the desired traits; Second, the most significant SNPs were extracted, which were identified by GWAS's construction on our dataset for each trait.

#### SNP selection based on previous findings

We have extracted associated recorded genes for HDL-C, LDL-C, TC, and TG, accessible on the GWAS Catalog database ( https://www.ebi.ac.uk/gwas)^5^. The entire SNPs were extracted within the identified genes and ± 10 kbp extended at both sides of the genes to control regulatory regions. Our findings comprised subsets of 15,910, 8796, 8935, and 14,158 SNPs within genes and 17,929, 10,299, 10,549, and 16,192 SNPs when extended ± 10 kbp at both sides of the genes included for HDL-C, LDL-C, TC, and TG, respectively. The detailed information for each trait is available in Supplementary File 1.xlsx.

#### SNP selection based on performing GWAS

According to this approach, after performing an association analysis, the SNPs were ranked based on their p-values. The SNPs were extracted from subsets of the top 1%, 5%, 10%, and 50%. These subsets contain 1%, 5%, 10%, 50% of the entire SNPs with the lowest p-value, respectively. Subsets of the top 1%, 5%, 10%, and 50% included 5464, 27,327, 54,641, and 273,213 SNPs, respectively. The procedure was carried out for HDL-C, LDL-C, TC, and TG.

#### Checking accuracy

10-repeated tenfold Cross-validation (CV) was conducted to evaluate the performance of the proposed approaches. In each repeat, we randomly divided individuals into ten subsamples. Each subsample was considered as the validation set and others as a discovery set. The process followed until every ten subsets were placed in the validation set for exactly one time. The SNPs' effect sizes, which were estimated based on the discovery set, were used to calculate individuals' whole-genome risk prediction in the validation set, which were not involved in estimating SNPs' effect sizes. The entire process was repeated ten times to reduce the variance of prediction accuracy. The evaluation was based on the correlation between genetic values and adjusted phenotypes (sex, age, and BMI). The average CV-correlation is the index to compare the performance of different subset selection strategies and the model with entire SNPs included. In addition, we have randomly selected an equal number of SNPs to form subsets in order to evaluate the performance of the corresponding selected subsets. The schematic workflow for the analysis step is summarized in Supplementary Fig. 3.

### Ethics approval and consent to participate

The local ethics committee approved this study at Research Institute for Endocrine Sciences; Shahid Beheshti University of Medical Sciences (Research Approval Code: 98104 & Research Ethical Code: *IR.SBMU.Endocrine.REC.1398.121*). In this study, all participants provided written informed consent for participating in the study. The research has been performed in accordance with the Declaration of Helsinki.

## Results

### Basic phenotypes information

Supplementary Table 1 contains the basic characteristics of participants for lipid profile traits. The number of observed phenotypes is slightly different, and the mean difference between men and women for BMI and phenotypes (HDL-C, LDL-C, TC, and TG) is significant (p < 0.001). Supplementary Table 2 represents the linear regression models’ results for the selected fixed covariates for HDL-C, LDL-C, TC, and TG. As it shows, it can be observed that all considered covariates (Age, BMI, and Sex) are significantly associated with traits.

### Prediction accuracy

**Figure 1:**
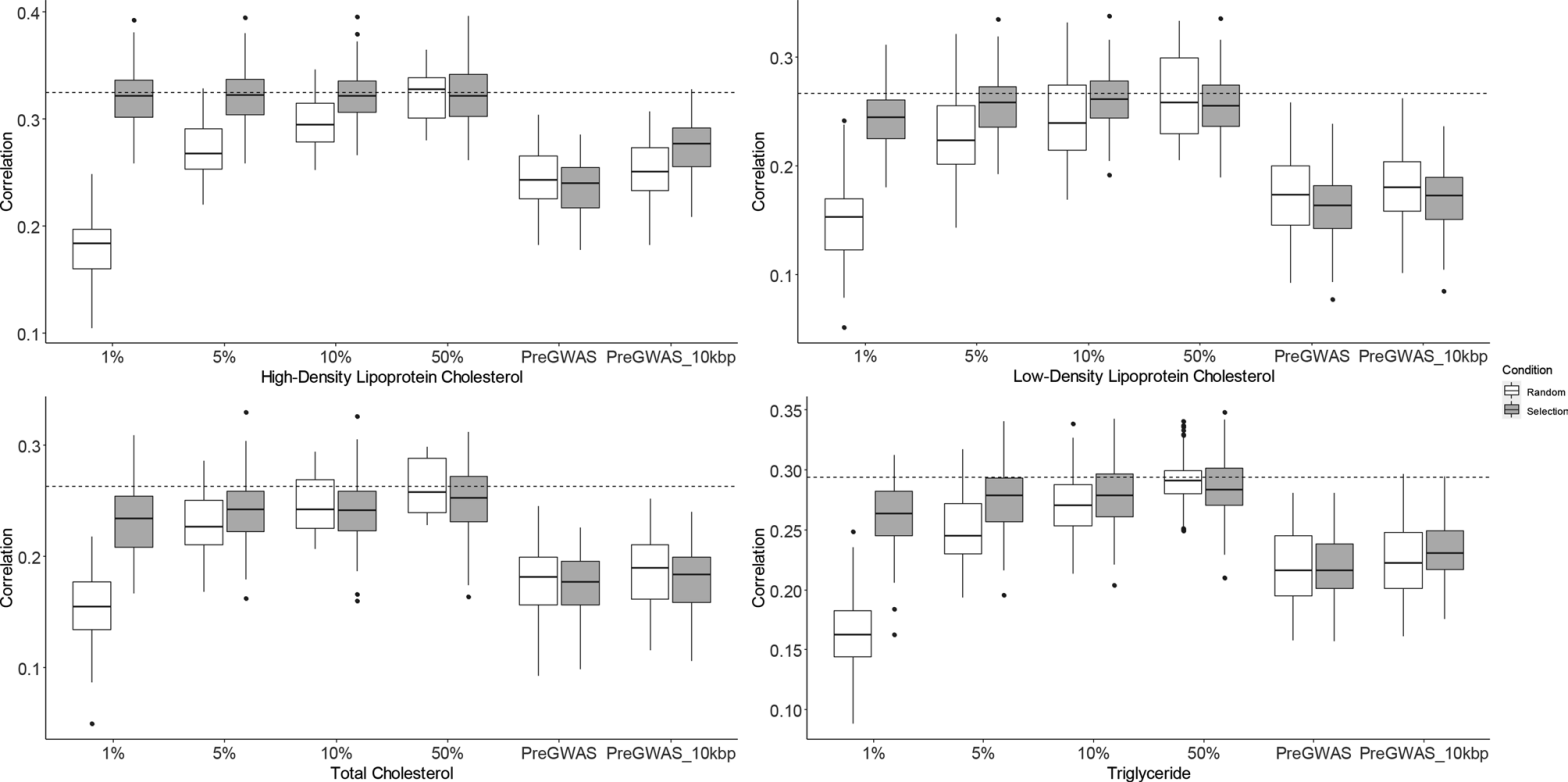
Distribution of CV-correlation between genetic prediction and adjusted phenotypes for HDL-C, LDL-C, TC, and TG. The average CV-correlation result based on tenfold 10-repeat between genetic prediction and adjusted phenotype (for age, sex, and BMI) is reported as the accuracy index. The dashed lines show the Prediction Accuracy obtained from the entire SNPs’ inclusion, which is almost the highest accuracy in most cases. The black boxes show the distribution of the tenfold 10-repeat cross-validation accuracy of selected SNPs based on proposed approaches. The gray boxes show the distribution of accuracy of the same cross-validation setting for the subsets of corresponding equally randomly selected SNPs. Subsets of the top 1%: 1% of the entire SNPs with the lowest p-value; Subsets of the top 5%: 5% of the entire SNPs with the lowest p-value; Subsets of the top 10%: 10% of the entire SNPs with the lowest p-value; Subsets of the top 50%: 50% of the entire SNPs with the lowest p-value; preGWAS: reported associated SNPs extracted from GWAS Catalog database; preGWAS_10kbp: within the identified genes and ± 10 kbp extended at both sides of the genes to control regulatory regions. All correlation coefficients were significant (< 0.000001). The highest prediction accuracy (dashed lines) was achieved when the entire SNPs were included for each trait. The lowest prediction accuracy was also achieved for each trait when subsets of associated SNPs from previous GWAS were used. For the first two subsets (1% and 5%), selected SNPs' accuracy is substantially more than random SNPs selected. Comparing the prediction in HDL-C, LDL-C, TC, and TG based on the GWAS subsets, the top 50% GWAS SNPs show the highest prediction accuracy.

The prediction accuracy for each lipid profile trait obtained from the gBLUP model using the entire SNPs and subsets of the top SNPs achieved from GWAS on our dataset (SNPs extracted to form subsets of the top 1%, 5%, 10%, and 50%) and subsets of SNPs based on the previous GWAS are visualized in Fig. 1. Here, the average CV-correlation result based on tenfold 10-repeat between genetic prediction and adjusted phenotype (for age, sex, and BMI) is reported as the accuracy index. All correlation coefficients in the two groups (selected and random groups for all six subsets) were highly significant (< 0.000001). The highest prediction accuracy (dashed lines) was achieved when the entire SNPs were included for each trait; HDL-C (r = 0.325), LDL-C (r = 4.16), TC (r = 0.260), and TG (r = 0.290). The lowest prediction accuracy was also achieved for each trait, HDL-C (r = 0.237), LDL-C (r = 0.162), TC (r = 0.175), and TG (r = 0.218) when subsets of associated SNPs from previous GWAS were used.

As Fig. 1 shows, selected subsets' accuracy is compared with randomly selected SNPs with an equal SNP number. The surprising result is that, in all traits, for the first two subsets (1% and 5%), selected SNPs' accuracy is substantially more than randomly selected SNPs. It demonstrates that the small number of large-effect SNPs' prediction accuracy is at least the same as all SNPs.

However, the accuracy of prediction increased as the number of SNPs in the subsets increased. Although the entire SNPs in each trait had the highest prediction accuracy, the differences between selected SNP subsets (the top 5%, 10%, 50% subsets) were comparatively small. Comparing the prediction in HDL-C, LDL-C, TC, and TG based on the GWAS subsets, the top 50% GWAS SNPs showed the highest prediction accuracy.

As is shown, roughly all selected SNPs based on GWAS subsets indicate more accuracy than randomly selected subsets except for the prediction accuracy difference on top 50% GWAS SNPs. At this point, randomly selected SNPs have better prediction accuracy than selected SNPs, but the difference is not significant. In roughly all traits, subsets with SNPs based on conducted GWAS showed significantly more prediction accuracy than subsets with SNPs based on previous GWAS. However, it may be due to the fact that previous GWAS subsets were less accurate than the conducted GWAS subset. For this reason, we compared the performance of conducted GWAS with an equal number of SNPs for each subset. On the other hand, the relatively high accuracy could be mainly due to using related individuals and existing patterns of overall relatedness and, consequently, existing relative patterns of linkage disequilibrium. It has been shown that genomic prediction models make better predictions using populations of related individuals with high linkage disequilibrium^9^.

### Annotation and genes

Of 546,339 SNPs, 56.94% were in the intronic region, 23.89% were in the intergenic region, and the other SNPs were in the rest of the annotated categories (downstream, exonic, non-coding, upstream, and UTR). In the Supplementary Table 3, we demonstrated the annotation of shared SNPs between each repeated fold (10-repeated tenfold) for different subset selections. It showed that almost half of the shared SNPs are in the intronic region of genes for each of the lipid profile traits, HDL-C (55.65% for top 1% SNPs, and 56.89% for top 50% of the SNPs), LDL-C (51.34% for top1% and 57.44% for top 50% of SNPs), TC (54.76% for top 1% of SNPs and 57.31% for top 50% of SNPs), and TG (54.74% for top 1% of SNPs and 57.48% for top 50% of SNPs). The second-highest number of the shared SNPs are in intergenic regions, as the annotation in the case of the top 1% of SNPs, is 18.34% for HDL-C, 22.63% for LDL-D, 18.33% for TC, and 18.95% for TG. However, the lowest number of SNPs are for the non-coding regions, as in HDL-C, the number of shared SNPs is 1.92% for the first subset (top 1%), 1.22% for LDL-C, 0.7% for TC, and 1.47% for TG.

For each trait, we selected the 100 most significant SNPs with a p-value from 1.45e−110 to 5.12e−06, in which some SNPs are common between traits. These variants for four traits included 306 unique SNPs and 81 related genes. Readers can find out more detailed information about these genes in Supplementary File 2.xlsx. Based on the GWAS catalog database^5^, they were associated with 2387 traits, and more than 50% of them (1244 traits) are reported to be associated with lipid profile traits.

### Heritability

**Figure 2:**
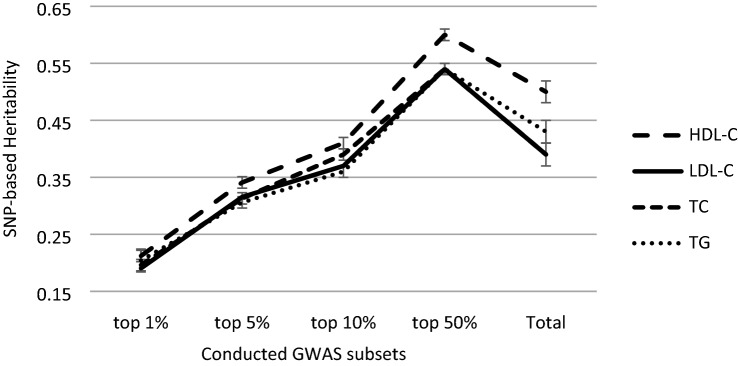
Heritability of lipid profile traits using the shared SNPs in the different repeated fold for different subset selection approaches. Heritability achieved by the shared top 50% approach has higher heritability than other subset selections (top 1%, 5%, and 10%) and than the total SNPs included.

Figure 2 shows the heritability obtained from shared SNPs between different repeated folds of selected SNPs in each approach (the number of shared SNPs are displayed for each approach in Supplementary Table 3). We found that the heritability achieved by the shared top 50% approach $\left({h}_{HDL-C\left(50\%\right)}^{2}=0.602,{h}_{LDL-C\left(50\%\right)}^{2}=0.544,{h}_{TC\left(50\%\right)}^{2}=0.542,{h}_{TG\left(50\%\right)}^{2}=0.544\right)$ has higher heritability not only compared to other subset selections (top 1%, 5%, and 10%) but also compared to the total SNPs included $({h}_{HDL-C(total)}^{2}=0.495,{h}_{LDL-C(total)}^{2}=0.388,{h}_{TC(total)}^{2}=0.390,{h}_{TG(total)}^{2}=0.431)$. Findings indicated that even though the number of SNPs used for heritability analysis was considerably low, heritability measures were relatively high.

## Discussion

This study investigated GWAS's incorporation in genomic prediction, applying the gBLUP method and gene-based heritability analysis on lipid profile traits (LDL-C, HDL-C, TC, and TG) using the genomic dataset of the Iranian population, TCGS project^14^. Recent studies have determined factors that affect the prediction accuracy of WGR, including (i) relatedness; the existence of relatives in testing and training data increases prediction accuracy^21^, (ii) traits' features; the more heritable the traits are, the better performance prediction is^22,23^, (iii) the genetic architecture of complex traits, e.g., the number of QTLs and their distributions^24,25^, (iv) LD between markers and QTLs; under perfect LD between markers and QTLs we can expect to approximately predict the full heritability of under-study traits^26^, (v) sample size; increasing the sample size can, possibly, close the gap between common SNP's heritability and the prediction R2^27,28^. However, the ability to catch more proportion of genetic variance explained by molecular markers is not necessarily translated into high prediction accuracy. For instance, a poor predictive ability for human height, as a trait with relatively high heritability^8^ achieved using genomic information^25^.

Many studies have compared various methods with different assumptions and different shrinkage approaches. Furthermore, Roudbar et al. showed that applying multi-omics data (integration of SNP markers and methylation sites) can increase the accuracy of the genomic prediction by comparing various methods^12^. We believe that controlling the factors mentioned above, which affect genetic prediction and are previously proven through previous studies, is very difficult. This is mainly due to the limitations and complex traits we are facing in human studies in practice. For these reasons, we tried to introduce a method to capture the most predictive SNPs, which is practical in most populations.

The high potential of GWAS findings in the clinical application, such as reported risk prediction, disease subtyping or classification, drug development, and drug toxicity^4^, encouraged scholars to apply association studies in prediction models, which is known as genetic risk score (GRS). GRS has shown promising results in the identification of high-risk individuals and families of CVD and dyslipidemia^29,30^, which variously forms from simplest versions, like allele count scores and weighted scores, to more sophisticated versions, including imputation^31,32^ and combining environmental and genetic effects^33^. Recently, researchers went further and tried to find predictive associated SNPs more meaningfully. For example, a conducted study on the Korean population selected the significant SNPs throughout the entire tenfold cross-validation sets to calculate weighted GRS on a discovery set^34^. Although their results on cholesterol ratios showed a good prediction accuracy, missing heritability is still an issue^6,8,35,36^, resulting in dismissing strong but not significantly associated SNPs. Motivated by this, we tried to introduce a method that benefits from the promising results of association studies and captures the possible genetic variation.

In this study, we used top SNPs based on the constructed GWAS results on our data set and previous studies. We showed that the conducted GWAS results on our dataset outperform the extracted associated SNP in previous studies. We assumed that this might be due to the different traits' genomic architecture; we can extract truly influential markers by performing GWAS on our dataset. The comparison of achieved results in prediction accuracy of the top SNPs ( 1, 5, 10, and 50 percent top SNPs) conveyed comparable prediction accuracy between the inclusion of subsets of the SNPs model and the inclusion of the entire, still statistically significant, SNPs model. Among subsets, selected top 50 percent SNPs in all traits showed the nearest prediction accuracy to the full models, which is due to the inclusion of a larger number of trait-related SNPs in the model. The importance of the number of SNP markers has already been investigated on HDL-C and LDL-C by comparing genetic prediction methods (from simple genetic risk score to different, more complex models) on a cohort study^37^. Helen Warren et al. concluded that the essential factor for the prediction model is the number of SNP markers included in the prediction model.

We found what we called “truly influential SNPs” by extracting shared SNPs in each repetition of performing GWAS, most of which were from the intronic region. The heritability of these subsets of SNPs showed interesting results. The relatively small number of SNPs in each strategy could capture marked genotypic variance. While including entire SNPs achieved gene-base heritability of 0.49, 0.388, 0.39, and 0.43 for HDL-C, LDL-C, TC, and TG, respectively, including the top 50 percent of SNPs achieved gene-based heritability of 0.602, 0.544, 0.542, and 0.544 for HDL-C, LDL-C, TC, and TG. These heritability improvements were not due to capturing the more genotypic variance, which increases inevitably by elevating the number of associated SNPs, but due to the reduction of the phenotypic error variance. In other words, reducing SNP markers to the most significant SNPs brings about capturing much of the genotypic phenotype variance and reducing the phenotypic error variance.

In summary, we cannot overlook the association studies' promising accomplishments in recent research regarding genomic prediction. However, including only, the statistically significant SNPs results in missing a great deal of information in genomic prediction and estimation of the gene-based heritability. We cannot expect to achieve much prediction accuracy by including significant SNPs based on previous studies. Investigating gBLUP accuracy on lipid profile traits showed that the top 1, 5, 10, and 50% SNPs based on constructed GWAS on our dataset achieved relatively accurate predictions. The highest prediction accuracy was achieved when comparatively more SNPs were involved. Analysis of gene-based heritability of lipid profile traits showed that we can capture almost all of the genotypic phenotype variance and reduce its error variance by including a subset of the mostly true trait-related SNPs.

This study only tested a single additive genetic variant method to find the most informative SNPs. In contrast, quantitative trait variability is commonly affected by multiple additive and non-additive sources such as epistatic interactions and dominant effects^38,39^. The utilization of statistical approaches that includes two-way interaction and dominant effects could lead to finding more informative SNPs to increase prediction accuracy, which can be found as a study topic for future research. Also, we suggest that other risk prediction methods can be used as a substitute for the gBLUP method, re-analyze our strategy, and compare their results with each other^40–44^.

## Supplementary Information

Supplementary File 1.

Supplementary File 2.

Supplementary Tables and Figures.

## Data Availability

The datasets used and analysed during the current study are available from the corresponding author on reasonable request.
