# Supplementary material for: GWAS findings improved genomic prediction accuracy of lipid profile traits: Tehran Cardiometabolic Genetic Study
Source: Sci Rep. 2021 Mar 11;11:5780. doi: 10.1038/s41598-021-85203-8 (PMC7952573; doi:10.1038/s41598-021-85203-8)
Supplement: Supplementary file 3 — Supplementary Tables and Figures. [file 41598_2021_85203_MOESM3_ESM.docx]

**Title:** GWAS findings improved genomic prediction accuracy of lipid profile traits: Tehran Cardiometabolic Genetic Study

**Running Title: Improve Genomic Prediction using GWAS**

**Author's name:**

1. Mahdi Akbarzadeh, Ph.D., Cellular, and Molecular Research Center, Research Institute for Endocrine Sciences, Shahid Beheshti University of Medical Sciences, Tehran, Iran. Email: ​akbarzadehms@sbmu.ac.ir
2. Saeid Rasekhi Dehkordi, MSc, Cellular and Molecular Research Center, Research Institute for Endocrine Sciences, Shahid Beheshti University of Medical Sciences, Tehran, Iran. Email: dehkordi.s.r@gmail.com
3. Mahmoud Amiri Roudbar, Department of Animal Science, Safiabad-Dezful Agricultural and Natural Resources Research and Education Center, Agricultural Research, Education & Extension Organization (AREEO), Dezful, Iran, Email: mahmood.amiri225@gmail.com
4. Mehdi Sargolzaei, Department of Pathobiology, Ontario Veterinary College, University of Guelph, Guelph, Canada, and Select Sires Inc., Plain City, USA, Email: msargol@uoguelph.ca
5. Kamran Guity, MSc, Cellular and Molecular Research Center, Research Institute for Endocrine Sciences, Shahid Beheshti University of Medical Sciences, Tehran, Iran. Email: kamran.guity@gmail.com
6. Bahareh Sedaghati-khayat, MSc, Cellular and Molecular Research Center, Research Institute for Endocrine Sciences, Shahid Beheshti University of Medical Sciences, Tehran, Iran. Email: [b.s.khayat@gmail.com](mailto:b.s.khayat@gmail.com)
7. Parisa Riahi, MSc, Cellular and Molecular Research Center, Research Institute for Endocrine Sciences, Shahid Beheshti University of Medical Sciences, Tehran, Iran. Email: parisaariyahii@gmail.com
8. Fereidoun Azizi, MD, Ph.D., Endocrine research center, Research Institute for Endocrine Sciences, Shahid Beheshti University of Medical Sciences, Tehran, Iran. Email: azizi@endocrine.ac.ir
9. Maryam S Daneshpour, Ph.D., Cellular and Molecular Research Center, Research Institute for Endocrine Sciences, Shahid Beheshti University of Medical Sciences, Tehran, Iran. Email: daneshpour@sbmu.ac.ir

**Correspondence Author:**

Maryam S Daneshpour, Cellular, and Molecular Research Center, Research Institute for Endocrine Sciences, Shahid Beheshti University of Medical Sciences, Tehran, Iran; POBox: 19195-4763, Tel: +982122432500, Fax: +982122416264, Email: daneshpour@sbmu.ac.ir

Supplementary Figure. 1: Genotype and samples QC: Quality control for samples is shown in green boxes, and for SNPs are shown in red boxes.

Input:
15000 individuals included
652919 SNPs

Pedigree Check:
15000 individuals

887 Samples excluded

Parentage Test:
325 individuals turned to founders

QC Input:
14113 individuals
 652919 Marker

Easy restriction:

SNPs missing<0.2
770 SNPs excluded

Sample missing <0.2
11 Samples were excluded

Rigid restriction:

SNPs missing<0.02
17636 SNPs excluded

Sample missing <0.02
No Samples were excluded.

MAF < 0.05
72500 SNPs excluded

HWE < 1e-6
1125 SNPs excluded

Sex Check
No Samples were excluded

Heterozygosity
317 samples excluded

Impute missing genotypes

Final dataset: 13785 Individuals
 546339 SNPs

Supplementary Figure. 2: After pruning for the (first/second) principal components via multi-dimensional scaling method


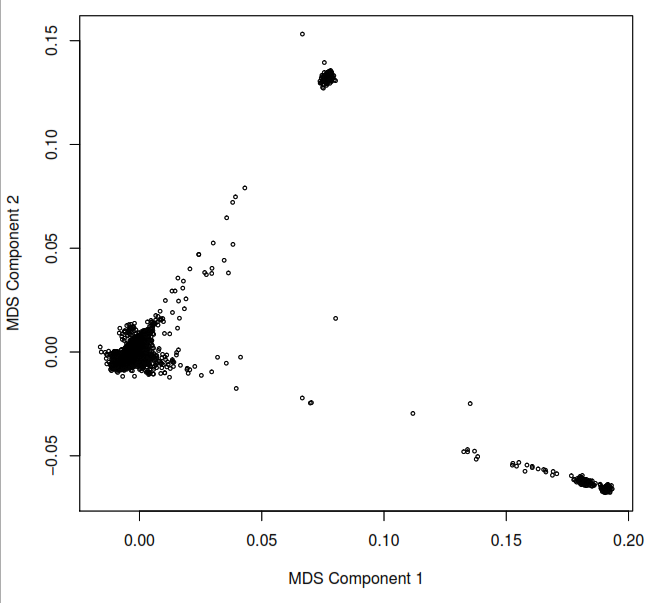


Supplementary Figure. 3: The workflow of analysis

Input: Total Number of SNPs
 Total Available Phenotypes

Subset Selection:
total Phenotypes + Selected SNPs

Discovery Set: contain 90 percent of phenotypes and selected SNPs

Validation Set: contain 10 percent of phenotypes and selected SNPs

Learning:

1st:

2nd:

3rd:

Making GRM

Genetic value calculated based on estimated SNP variance using REML

Genetic value for individual converts to SNPs effect size

Genetic value for validation set calculated using estimated SNPs effect size from discovery set

Correlation between adjusted Phenotypes and Genetic values calculated

Supplementary Table 1: Basic characteristics of participants (standard errors) for the traits under study.

|  | HDL-C (n=10301) | | | LDL-C (n=10301) | | | TC (n=10301) | | | TG^*^ (n=10301) | | |
| --- | --- | --- | --- | --- | --- | --- | --- | --- | --- | --- | --- | --- |
|  | **Men**  )4683( | **Women**  )5618( | **Pvalue** | **Men**  )4790( | **Women**  )5796( | **Pvalue** | **Men**  )4686( | **Women**  )5617( | **Pvalue** | **Men**  )4686( | **Women**  )5617( | **Pvalue** |
| Age Mean(SD) | 40.00  (19.00) | 39.72  (17.79) | 0.41 | 40.31  (19.72) | 40.22  (18.20) | 0.82 | 40.02  (19.42) | 39.72  (17.79) | 0.40 | 40.02  (19.42) | 39.72  (17.79) | 0.40 |
| BMI  Mean(SD) | 25.91  (5.00) | 27.47  (5.89) | <0.001 | 25.33  (6.25) | 26.64  (7.66) | <0.001 | 25.92  (5.00) | 27.47  (5.89) | <0.001 | 25.92  (5.00) | 27.47  (5.89) | <0.001 |
| Phenotype  Mean(SD) | 43.91  (10.11) | 50.95  (11.20) | <0.001 | 108.62  (33.25) | 109.49  (34.35) | 0.18 | 181.16  (39.31) | 185.59  (41.00) | <0.001 | 4.83  (0.54) | 4.70  (0.53) | <0.001 |

*A log transformation was applied to adjust the skewed distribution of TG.

As it shows, the number of participants with High-Density-Lipoprotein (LDL-C) is 4683 (men) and 5618(women), with the mean age of 40.03 (SE=19.42) and 39.72(SE=17.79), respectively. There was a significant difference in BMI ranges for this group that has been measured to be 25.91(SE=5.00) for men and 27.47(SE=5.89) for women (P-value=2.2×10^-6^). However, 4790 men and 5796 women with LDL-C have been included in the study with the mean age of 40.31(SE=19.72) and 40.22 (SE=18.20), respectively. According to the P-value provided, the significant difference between the BMI ranges in the two groups was proven. Also, male participants were 4686 in the TC group, and the female was 5617, with a mean age of 40.02 (SE=19.42) and 39.72 (SE=17.79), respectively. Their BMI ranges differed significantly between men (25.92, SE=5.00) and women (27.47, SE=5.89). As is shown, 4686 males and 5617 females in the triglyceride group with the mean age of 40.02 (SE=19.42) and 39.72 (SE=17.79), and with significant BMI ranges (P-value=2.2×10^-16^, men with 25.92 (SE=5.00) and women with 27.47 (SE=5.89)) have participated in the study.

Furthermore, except for LDL-C (P-value=0.1849), other phenotypes ((HDL-C, P-value=2.2×10^-6^), (TC, P-value=2.33×10^-8^), TG, P-value=2.2×10^-16^)) significantly differed between men and women. The mean of phenotypes for HDL-C was 40.03 (SE=19.42) for males and 39.72 (SE=17.79) for women, and for LDL-C was 108.62 (SE=33.25) for male and 109.49 (SE=34.35) for female. The mean of observed phenotype for the TC and TG were 181.16 (SE=39.31) and 4.83 (SE=0.54) for men, and 185.59 (SE=41.00) and 4.70 (SE=0.53) for women, respectively.

Supplementary Table 2: The estimated parameters form the linear model with age, BMI, and sex as covariates for HDL-C, LDL-C, TC, and TG.

| HDL-C | | LDL-C | | TC | | TG^*^ | |  |
| --- | --- | --- | --- | --- | --- | --- | --- | --- |
|  | **N=10301** | | **N=10586** | | **N=10303** | | **N=10303** | |
|  | Estimate (S.E) | P-value | Estimate (S.E) | P-value | Estimate (S.E) | P-value | Estimate (S.E) | P-value |
| Age (years) | 0.012 (0.006) | 0.058 | 0.54(0.016) | <0.001 | 0.68 (0.02) | <0.001 | 0.01(0.001) | <0.001 |
| BMI (kg/m^2^) | -0.503 (0.020) | <0.001 | 0.54 (0.045) | <0.001 | 1.06 (0.073) | <0.001 | 0.03 (0.001) | <0.001 |
| Sex | 7.82 (0.207) | <0.001 | 0.21 (0.623) | 0.739 | 2.98 (0.73) | <0.001 | -0.17 (0.01) | <0.001 |

^*^ A log transformation was applied to adjust the skewed distribution of TG.

Still, there is no significant relationship between sex and LDL-C level. Between the age and HDL-C levels, as the P-value for the Age (0.012(0.01)), BMI (-0.50(0.02)), and sex (7.82(0.20)) against HDL-C are 0.0583,<2.2×10-16, 2.2×10-16 and the corresponding coefficients against LDL-C are 0.54(±0.01683) (P-value <2.2×10-16), 0.54(0.05) (P-value <2.2×10-16), and 0.21(0.62) (P-value=0.739). As is shown, the regression coefficients of the covariates and TC (Age 0.68(0.02) P-value <2.2×10-16, BMI 1.06(0.07) P-value <2.2×10-16 and Sex 2.98(0.73) P-value=5.38×10-5 ) are significant. Considering the log transformation of triglyceride, all of the covariates, Age (0.01(0.001)), BMI (0.03 (0.001)), and sex (-0.17±0.01), with the P-value <2.2×10-16 were significant.

Supplementary Table 3. Annotation of shared SNPs between different repeated fold for different strategies in lipid profile traits

|  |  | downstream | Exonic | intergenic | Intronic | non-coding | upstream | UTR | Sum |
| --- | --- | --- | --- | --- | --- | --- | --- | --- | --- |
| HDL-C | 1% | 26(5.54%) | 23(4.9%) | 86(18.34%) | 261(55.65%) | 9(1.92%) | 40(8.53%) | 24(5.12%) | 469 |
|  | 5% | 207(6.08%) | 127(3.73%) | 743(21.84%) | 1920(56.44%) | 47(1.38%) | 254(7.47%) | 104(3.06%) | 3402 |
|  | 10% | 524(6.18%) | 252(2.97%) | 1949(22.99%) | 4776(56.35%) | 130(1.53%) | 618(7.29%) | 227(2.68%) | 8476 |
|  | 50% | 5212(6.27%) | 2073(2.49%) | 19862(23.9%) | 47278(56.89%) | 1258(1.51%) | 5468(6.58%) | 1957(2.35%) | 83108 |
| LDL-C | 1% | 28(6.81%) | 25(6.08%) | 93(22.63%) | 211(51.34%) | 5(1.22%) | 33(8.03%) | 16(3.89%) | 411 |
|  | 5% | 216(6.75%) | 96(3%) | 748(23.39%) | 1791(56%) | 42(1.31%) | 222(6.94%) | 83(2.6%) | 3198 |
|  | 10% | 547(6.79%) | 224(2.78%) | 1826(22.66%) | 4620(57.33%) | 119(1.48%) | 513(6.37%) | 210(2.61%) | 8059 |
|  | 50% | 5053(6.21%) | 2021(2.48%) | 19138(23.5%) | 46770(57.44%) | 1206(1.48%) | 5206(6.39%) | 2028(2.49%) | 81422 |
| TC | 1% | 30(6.96%) | 30(6.96%) | 79(18.33%) | 236(54.76%) | 3(0.7%) | 39(9.05%) | 14(3.25%) | 431 |
|  | 5% | 219(7.06%) | 109(3.51%) | 694(22.38%) | 1732(55.85%) | 38(1.23%) | 221(7.13%) | 88(2.84%) | 3101 |
|  | 10% | 531(6.75%) | 244(3.1%) | 1839(23.38%) | 4437(56.41%) | 101(1.28%) | 513(6.52%) | 200(2.54%) | 7865 |
|  | 50% | 4963(6.21%) | 2031(2.54%) | 18973(23.74%) | 45792(57.31%) | 1181(1.48%) | 5004(6.26%) | 1964(2.46%) | 79908 |
| TG | 1% | 44(7.19%) | 28(4.58%) | 116(18.95%) | 335(54.74%) | 9(1.47%) | 60(9.8%) | 20(3.27%) | 612 |
|  | 5% | 224(7.02%) | 114(3.57%) | 658(20.62%) | 1781(55.81%) | 58(1.82%) | 255(7.99%) | 101(3.17%) | 3191 |
|  | 10% | 518(6.53%) | 236(2.97%) | 1751(22.07%) | 4502(56.75%) | 136(1.71%) | 557(7.02%) | 233(2.94%) | 7933 |
|  | 50% | 5005(6.24%) | 2017(2.52%) | 18612(23.21%) | 46099(57.48%) | 1219(1.52%) | 5174(6.45%) | 2068(2.58%) | 80194 |
| Entire SNPs | | 27235(4.98%) | 14607(2.67%) | 130554(23.89%) | 311113(56.94%) | 7817(1.43%) | 41085(7.52%) | 13928(2.54%) | 546339 |
